# Inferring Noncoding RNA Families and Classes by Means of Genome-Scale Structure-Based Clustering

**DOI:** 10.1371/journal.pcbi.0030065

**Published:** 2007-04-13

**Authors:** Sebastian Will, Kristin Reiche, Ivo L Hofacker, Peter F Stadler, Rolf Backofen

**Affiliations:** 1 Bioinformatics Group, Institute of Computer Science, University of Freiburg, Freiburg, Germany; 2 Bioinformatics Group, Department of Computer Science, University of Leipzig, Leipzig, Germany; 3 Department of Theoretical Chemistry, University of Vienna, Vienna, Austria; University of California San Diego, United States of America

## Abstract

The RFAM database defines families of ncRNAs by means of sequence similarities that are sufficient to establish homology. In some cases, such as microRNAs and box H/ACA snoRNAs, functional commonalities define classes of RNAs that are characterized by structural similarities, and typically consist of multiple RNA families. Recent advances in high-throughput transcriptomics and comparative genomics have produced very large sets of putative noncoding RNAs and regulatory RNA signals. For many of them, evidence for stabilizing selection acting on their secondary structures has been derived, and at least approximate models of their structures have been computed. The overwhelming majority of these hypothetical RNAs cannot be assigned to established families or classes. We present here a structure-based clustering approach that is capable of extracting putative RNA classes from genome-wide surveys for structured RNAs. The LocARNA (local alignment of RNA) tool implements a novel variant of the Sankoff algorithm that is sufficiently fast to deal with several thousand candidate sequences. The method is also robust against false positive predictions, i.e., a contamination of the input data with unstructured or nonconserved sequences. We have successfully tested the LocARNA-based clustering approach on the sequences of the RFAM-seed alignments. Furthermore, we have applied it to a previously published set of 3,332 predicted structured elements in the Ciona intestinalis genome (Missal K, Rose D, Stadler PF (2005) Noncoding RNAs in *Ciona intestinalis.* Bioinformatics 21 (Supplement 2): i77–i78). In addition to recovering, e.g., tRNAs as a structure-based class, the method identifies several RNA families, including microRNA and snoRNA candidates, and suggests several novel classes of ncRNAs for which to date no representative has been experimentally characterized.

## Author Summary

For a long time, it was believed that the control of processes in living organisms is almost only performed by proteins. Only recently, scientists learned that a further class of molecules, namely special RNAs, plays an important role in cell control. In consequence, research on such RNAs enjoys increasing attention over the last few years. These RNAs were called noncoding RNAs (ncRNA), because, unlike most other RNAs, these molecules do not code for proteins. Due to recent research successes, one can predict a lot of potential new ncRNAs by comparing the genomes of related organisms. Technically, comparing such RNAs is challenging and computationally expensive, since related ncRNAs often show only weak similarity on the sequence level, but share similar structures. In the paper, we present the new method LocARNA for fast and accurate comparison of RNAs with respect to their sequence and structure. Using this method, we define a distance measure between pairs of ncRNAs based on sequence and structure. This is then used for combining RNAs into a cluster for identifying groups of similar RNAs in large unorganized sets of RNA. The final aim of such a comparison is to identify new classes of ncRNAs. We applied our clustering procedure to a previously published set of 3,332 predicted ncRNAs in the C. intestinalis genomes. In addition to rediscovering known classes of RNAs, e.g., tRNAs, the method predicts microRNA candidates, and suggests several novel, experimentally uncharacterized classes of ncRNAs. For verification, we clustered about 4,000 RNAs of RFAM, which is a large database that contains RNAs with an already known classification into families. Our results show good performance of the presented structure-based clustering approach.

## Introduction

Starting with the discovery of microRNAs [1–3] and the advent of genome-wide transcriptomics [4–6], it has become obvious that RNA plays a large variety of important, often regulatory, roles in living organisms that extend far beyond being a mere intermediate one in protein biosynthesis. The elucidation of the functional roles of the plethora of newly discovered ncRNAs has thus become a central research interest in molecular biology.

Recent advances in computational RNomics have resulted in numerous software packages that can be employed to detect ncRNAs with evolutionarily conserved secondary structures [7–12]. Two of these, EvoFold [10] and RNAz [9,13], are efficient enough to be applied to genome-wide surveys in mammals [10,13] and other metazoan clades [14,15]. Both approaches start from multiple sequence alignments. While EvoFold uses the SCFG approach pioneered by qrna [7], RNAz is based on evaluating the folding thermodynamics. Both approaches classify input alignments either as unstructured or as possessing a common RNA secondary structure; in the latter case they provide a prediction for the consensus structure of the aligned sequences.

Just as in the case of proteins, ncRNA sequences can be grouped into families that are characterized by clear homologies. Usually the members in a family share functional characteristics as well as conserved sequence and structure motifs. Indeed, the RFAM database [16] compiles several hundred families of ncRNAs based on this observation. Examples include the individual snRNAs U1, U2, U4, U5, and U6, 5S rRNA, RNAse P RNA, the RNA component of telomerase, more than a hundred families of snoRNAs, and several hundred microRNA families collected in mirbase [17]. In many cases, RNA families can be grouped together, forming a ncRNA class whose members have no discernible homology at sequence level, but still share common structural and functional properties. The best-known classes are tRNAs (although it is well-established that all tRNAs derive from a common ancestor [18]), the two distinct classes of snoRNAs (box H/ACA and box C/D), RNAse P and MRP RNAs, and microRNAs. It is thus natural to ask whether the many ncRNA candidates that have been predicted computationally can be grouped into families or even classes, and in particular, whether there is evidence for novel families and classes for which we have not yet seen experimentally verified representatives.

As sequence similarity is often remote even within well-established RNA families, we cannot rely on pure sequence alignment techniques for this task. Indeed, it has been shown that sequence alignments of structured RNAs fail at pairwise sequence identities below about 60% [19]. Several different algorithmic approaches have been introduced in the past to determine structural similarities and to derive consensus structure patterns for RNAs that are too diverse to be alignable at sequence level. The corresponding software tools, such as MARNA [20], PMmulti [21], and RNAforrester [22] cannot be applied without modifications to the problem of clustering predicted structures from RNAz or EvoFold surveys, however. The main reason is that these ncRNA detectors are not guaranteed to find the complete ncRNA genes; rather they usually detect particularly conserved substructures and sometimes the predictions are contaminated with spurious predictions in the flanking sequences. Thus, a local structure-based alignment algorithm is necessary. This is already implemented in RNAforrester [22], which is based on tree-alignment, and in the local sequence–structure alignment approach described [23], which in addition can also detect structurally local motifs. A related approach detects exact local sequence structure patterns in **O**(*n* ^2^) [24]. However, all these approaches require a single known or predicted input structure. Tree-alignment and tree-editing in addition have only limited capabilities to repair incorrect base pairs. Tree-alignment is particularly restrictive in this respect since even broken arcs must be nested. As a consequence, RNAforrester tends to produce many alignment columns that contain mostly gap characters in the multiple alignment mode.

In contrast, derivates of the Sankoff algorithm [25] solve the problem of simultaneous folding and alignment, which turned out to be more appropriate. However, the large number of predicted ncRNAs, several thousands in the case of nematode and urochordate genomes and close to 100,000 in the case of mammals, calls for more efficient variants of these algorithms.

In this contribution we introduce LocARNA (local alignment of RNA), a local pairwise structural alignment algorithm for pseudoknot-free RNA secondary structures, and its multiple version mLocARNA. (m)LocARNA is a Sankoff-style algorithm, similar to PMmulti, that is efficient enough to be used for large clustering of predicted ncRNAs. We have successfully tested the LocARNA-based clustering approach on the sequences of the RFAM-seed alignments to demonstrate the feasibility of the approach, and to evaluate the results. Furthermore, we use the data from a survey of the ascidians C. intestinalis and C. savignyi [14] to achieve the following goals. (1) We search for novel, clade-specific RNA families in *Ciona,* which is of interest in itself. (2) In doing so, we can increase the credibility of some of the predicted ncRNAs, since being part of larger family of related RNAs with similar structure reduces the likelihood of being a false positive prediction. (3) We improve the genome annotation by assigning additional ncRNAs to known families. (4) The inferred consensus structures of novel families form a starting point for subsequent searches in related organisms.

## Methods

### Structure-Based Clustering

In this work, we set up a pipeline for automated clustering of ncRNAs (or ncRNA candidates) and semi-automated selection of novel, complex clusters of RNAs. The input is a set of RNAs *R*_1_,…,*R*_N_, which are given by their sequences, and the output is a hierarchical clustering of these RNAs. In addition, we will generate a fast, presorted, and annotated overview of the clusters for further inspection by an expert. Our pipeline is built from the following steps:

1. For each of the RNAs, we compute structural information using McCaskill's algorithm, implemented in RNAfold. This algorithm computes a matrix of pair probabilities based on a complete energy model of RNAs.

2. The next step is to compute all pairwise alignments of the structurally annotated sequences using LocARNA. Note that this requires computation of **O**(*N* ^2^) pairwise sequence–structure alignments for determining the distance matrix. Note further that performing all pairwise comparisons cannot be reasonably circumvented or replaced in a full-featured clustering procedure. For genomic-scale datasets, **O**(*N* ^2^) comparisons are way too costly for most existing sequence–structure approaches. The computational efficiency remains crucial, even if this computationally most intensive procedure is distributed for parallel computation, which we do in a straightforward manner. As a result, we assign a LocARNA-alignment score (*i*,*j*) to each pair of RNAs (*R_i_*,*R_j_*).

3. A cluster-tree is generated by applying the weighted pair group method algorithm (WPGMA), which is also known as average-linkage clustering, to a matrix of pairwise distances of the RNAs. There, the distances *d*(*i*,*j*) correspond directly to our LocARNA-scores. Instead of computing distances as *max_ij_-score*(*i,j*)*,* we define distances by

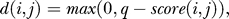
where *q* is the *x*-quantile (e.g., *x* = 99%) of all pairwise scores. This decision avoids the fact that exceptionally large scores influence the distance-transformation. In the resulting tree, internal nodes correspond to clusters of RNAs. Their heights correspond to the mean pairwise LocARNA-scores of their constituents and thus give a single-value measure of cluster quality.

4. A good overview and a true quality assessment of the clusters can be best provided through multiple alignments of each cluster. We simultaneously construct all multiple sequence–structure alignments, i.e., one for each cluster, by only **O**(*N*) runs of the pairwise alignment algorithm. This can be done by constructing the multiple alignments progressively, using the already constructed cluster-tree as guide tree.

From each of the multiple alignments, we collect information that can guide a quality assessment of the cluster. We compute the mean pairwise sequence identity (MPI) and, using RNAalifold, the structure conservation index (SCI), the consensus minimum free energy (MFE), the consensus MFE structure, and the consensus base pair probabilities. Sorting the list of generated clusters by the quantities size of cluster, SCI, MPI, and MFE provide the expert with an automatically proposed order for his manual inspection of the clusters. The multiple alignment itself and the consensus structure information facilitate the selection of “interesting” clusters.

This pipeline crucially depends on pairwise sequence–structure alignments. Therefore, we require the following algorithmic components which we describe in some detail in the next two sections, (1) LocARNA: Efficient Pairwise Local Sequence–Structure Alignment and (2) Local Multiple Sequence Structure Alignments. In (1), we develop an efficient algorithm for high-quality pairwise alignments of RNAs that considers both sequence and structure information. For this purpose, the best results are achieved with Sankoff-style algorithms. We provide the new method called LocARNA, which is much more efficient than current approaches and uses base pair probabilities as structural input. Regarding (2), for the selection of clusters, one important subproblem is to extract consensus structure information from the clustered RNAs, which is done by using RNAalifold. For producing the input for RNAalifold, we introduce the *local* multiple alignment method mLocARNA.

### LocARNA: Efficient Pairwise Local Sequence–Structure Alignment

For the pairwise alignments of RNA, we use our novel tool LocARNA, which computes local alignments of RNA. It is a Sankoff-style algorithm in the spirit of PMcomp, but goes beyond its ancestor by introducing local alignment and significantly improving the efficiency.

The Sankoff algorithm [25] provides a general solution to the problem of simultaneously computing an alignment and the common secondary structure of the two aligned sequences. In its full form, the problem requires **O**(*n* ^6^) CPU time and **O**(*n* ^4^) memory, where *n* is the length of the RNA sequences to be aligned. In general, one can distinguish two variants of the Sankoff algorithms: programs such as foldalign [26,27] and dynalign [28] implement a more or less complete energy model for the RNA folding part. In contrast, PMcomp [21] assumes that a structure model for the two input sequences is already known and given in the form of weights for the individual base pairs. However, note that such a structure model is reasonably obtained using McCaskill's algorithm [29], again on the basis of a full-featured energy model.

Consider two sequences *A* and *B* with associated base pair probability matrices *P^A^* and *P^B^*, respectively. The goal is to compute a sequence alignment **A** of *A* and *B* together with secondary structure **S** on **A**. **A** consists of a set of (mis)matches written as pairs (*i*,*k*), where *i* is a position in *A,* and *k* a position in *B*. The consensus secondary structure **S** for an alignment **A** consists of a set of quadruples (*ij*;*kl*), where (*i*,*k*)∈**A** and (*j*,*l*)∈**A** are two matches in **A**, (*i*,*j*) is a base pair on sequence *A,* and (*k*,*l*) is a base pair on sequence *B*. Furthermore, denote by **A** *_s_* the single-stranded part of the alignment; i.e., if (*i*,*k*)∈**A** *_s_* then there is no pair (*j*,*l*) such that (*ij*;*kl*)∈**S** or (*ji*;*kl*)∈**S**. The goal is to determine the pair (**A**,**S**) that maximized the score function

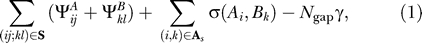
where 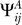 and 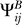 are base pair scores (see below), σ:{*A*,*C*,*G*,*U*}^2^ → **R** is the similarity score for (mis)matches, γ is the gap score parameter, and *N*_gap_ is the number of insertions and deletions in the alignment **A**.

Both PMcomp and our novel tool, LocARNA, use base pair scores that are derived from the base pairing probability matrices of the two individual sequences. More precisely, we use here

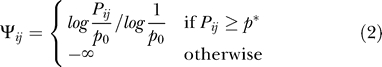
where *P_ij_* is the equilibrium pairing probability as computed by McCaskill's algorithm [29], *p*_0_ is the expected probability for a pairing to occur at random, and *p* ^*^ is the cutoff probability, below which the arcs are ignored. Formally, this is expressed by assigning −∞ as the weight in this case. The term 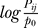 is the log-odds score for having a specific base pairing against the null model of a random pairing, and 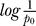 is a normalization factor that transforms the weights to a maximum of 1. The reason for this normalization is just that it is easier to balance the sequence score against the structure score.

LocARNA improves the PMcomp approach in several ways. First of all, it uses a modified dynamic programming approach that allows us to utilize the fact that typically the number of significant base pairs does not grow with **O**(*n* ^2^), i.e., that the base pair probability matrices *P^A^* and *P^B^* are usually sparse. In particular, if *p* is constant for different *n*, then each base can take part in at most 1/*p* ^*^, and thus **O**(1) base pairs. Hence, there are only *m* = **O**(*n*) significant entries in *P*.

We define *D_ij_*_;*kl*_ as the maximal similarity score of an alignment for the subsequences *A*[*i*..*j*] and *B*[*k*..*l*] with the additional condition that (*i j*;*k l*) is part of consensus secondary structure. To profit from the reduced number of significant base pairs in time and space complexity, we calculate and store only *D_ij_*_;*kl*_ that correspond to significant base pairs. Due to this modification, we need to take special care to avoid redundant computation. Therefore, we compute the entries *D_ij_*_;*kl*_ by fixing *i* and *k* and varying only *j* and *l*. We introduce the notation *D_i•_*_;*k•*_ to denote the matrix slice where *i* and *k* are fixed. The efficient calculation of *D_i•_*_;*k•*_ in **O**(*n* ^2^) time requires an auxiliary matrix *M*, where the entries *M_ij_*_;*kl*_ are the optimal similarity score of subsequences *A*[*i* + 1…*j*] and *B*[*k* + 1…*l*], and leads to a computation order that differs from PMcomp. Finally, the dynamic programming recursion for *M* and *D* takes the usual form of a Sankoff-style algorithm:

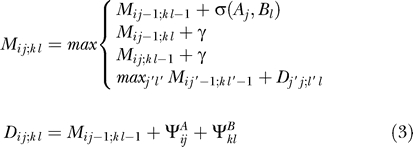



The important observation is that the last, computationally most expensive, alternative in the *M* recursion needs to be evaluated only for *P^A^_j_*_′*j*_ ≥ *p* ^*^ and *P^B^_l′l_* ≥ *p* ^*^, and, analogously, *D* needs to be stored only for matching base pairs. We observe that *D*_*i*•__;*k*•_ depends only on *M_i•_*_;*k•*_, which in turn can be computed from other *M_i•_*_;*k•*_ entries. Thus, we only need to store the entries of *M* for the current values of *i* and *k*, i.e., **O**(*n* ^2^) entries. The recursion can therefore be evaluated in **O**(*m* ^2^ + *n* ^2^) memory and **O**(*n* ^2^(*n* ^2^ + *m* ^2^)) time.

From the matrices *M* and *D,* we can now compute the score of the best global alignment as well as the score of the best local alignment. In our study, we are only interested in the latter. Global alignment is only explained for better understanding and for comparison to the global alignment algorithm PMcomp. The score of the global alignment can be computed by evaluating the recursion for *M*_0*j*;0*l*_, i.e., the optimal global alignment score is *M*_0|*A*|;0|*B*|_.

Recall that our main goal is to apply the procedure to the prediction of ncRNA detectors (e.g., such as RNAz) as generated by genome-wide screens. These detectors are not guaranteed to find the complete ncRNA genes and usually detect conserved substructures. Moreover, the predictions can be contaminated with spurious predictions in the flanking sequences. Hence, we need local sequence–structure alignment.

Concerning local alignment, in a Sankoff-style approach usually we compute a four-dimensional matrix of alignment scores for *each* pair of subsequences *A*[*i…j*] and *B*[*k…l*]. In this case, we could trivially obtain the best local alignment score by searching for the maximal score.

In our case, however, we cannot apply this simple method, since we do not compute entries for all possible pairs of subsequences. Rather, we compute only scores for subsequences that are closed by (significant) base pairs or prefixes of them. Those scores are either stored in *D_ij_*_;*kl*_ (in the case of closing a base pair match) or in *M_ij_*_;*kl*_.

Instead, we will borrow, slightly tailored for our purpose, the trick of standard sequence alignment, which is to add an additional zero entry in the recursion for cutting off dissimilar prefix-alignments. Note that this assumes that the score parameters yield a score greater than zero only for similar subsequences. The best local alignment is then obtained as the maximal entry of the matrix.

However, note that we must not change the recursion equations for all *M_ij_*_;*kl*_, which serve for computing some entry of *D*. Only for alignments of subsequences *A*[*i…j*] and *B*[*k…l*], where at least one of the subsequences is not enclosed by a (significant) base pair, is it correct to cut off dissimilar prefix-alignments. All these cases are accounted for when considering the alignments of all pairs of prefixes of *A* and *B*, which are stored in the *M*_0•;0•_ slice.

Therefore, we use for local alignment the following variant of Equation 3 that extends Equation 3 only for the slice *M*_0•;0•_.

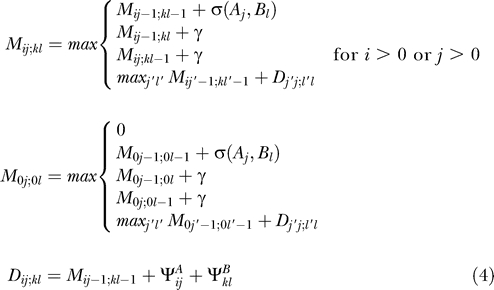



Note that the entries *M*_0*j*;0*l*_ will not be needed to compute any entry *D_i_*_′*j*′;*k*′*l*′_. By adding a 0-entry in the calculation of *M*_0*j*;0*l*_, we ensure that entries in *M*_0•;0•_ are nonnegative. Since negative scores are considered dissimilar, we thereby remove prefix-alignments that do not belong to the local alignment. The optimal local alignment score is then max*_jl_*(*M*_0*j*;0*l*_).

The corresponding optimal alignment and consensus secondary structure can now be obtained by backtracing, i.e., for local alignment we start from the maximal entry in *M*_0*j*;0*l*_ and stop when similarity drops to its minimal value of zero. In addition, for every pair (*i j*;*k l*) in the consensus structure we have to recompute the *M_i_*_•;*k*•_ at a cost of **O**(*n* ^2^+*m* ^2^). Since there are at most **O**(*n*) pairs in the consensus structure, the cost of backtracing stays negligible.

LocARNA is implemented in C++, which results in a further performance gain relative to the Perl implementation of PMcomp. While it fully exploits speed and memory reductions that can be obtained by limiting possible consensus structures, additional performance gains are possible by restricting the possible sequence alignments. This is done, e.g., in stemloc [30] by using “alignment envelops”. A similar but more easily implemented technique is used by CONSAN [31], where high confidence matches (“pins”) are derived from local sequence alignments. The algorithm then considers only alignments that contain all pins.

### Local Multiple Sequence–Structure Alignments

Based on the pairwise LocARNA algorithm, we construct a progressive multiple alignment method, mLocARNA, which is similar in spirit to PMmulti, the PMcomp-derived multiple alignment tool [21]. mLocARNA differs from PMmulti in the algorithm for computing the consensus base pairing probability matrix *P^A∘B^* for the combined alignment of *A* and *B* from the base pairing probability matrices of the subalignments (or sequences) *A* and *B*. For a pair of columns *p, q* in the alignment of *A* and *B*, PMcomp defines the combined base pair weight by

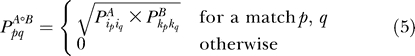
where *i_p_* and *i_q_* are the positions corresponding to *p* and *q* in the subalignment *A,* respectively. *k_p_* and *k_q_* are defined analogously for subalignment *B*. This has the effect that whenever one subalignment contains a gap at *p* or *q* or has a very low base pair probability, then the structural information between *p* and *q* from the other subalignment is effectively lost. In consequence, PMmulti tends to remove most base pairs when aligning many sequences.

To avoid this undesired effect, we introduce the new definition

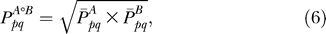
where

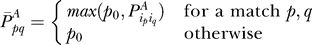
and 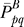 is defined analogously.

As usual, the order of pairwise alignments is directed by a guide tree. We use for that purpose the sub-trees produced by the hierarchical clustering.

## Results

### Evaluation of the Clustering Procedure

To evaluate the quality of our clustering approach, we have applied our procedure to the sequences in the RFAM seed alignments. Our test set consists of all seed sequences that have no more than 80% sequence identity and do not exceed 400 nt in length, resulting in 3,901 sequences from 503 families. Normally, quality measures such as sensitivity and specificity are defined for binary classification problems, while here we face the problem of comparing our hierarchical clustering with the family assignment in RFAM. In principle, there are two ways of looking at the problem, namely globally (considering the complete set of clusters), and locally (considering the quality for each family separately).

**Figure 1:**
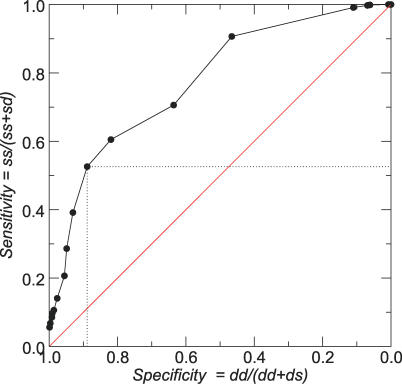
ROC Curve of the Global Comparison of Clustering and RFAM Families At a false positive rate of 12%, we achieve a sensitivity of 52% (correctly grouping together sequences of the same family), which is more than sufficient to detect families.

Concerning the global view, the complete RFAM defines a partition of the set of all sequences into families (or clusters), and we can compare the degree of agreement between the partition defined by our clustering with the partition defined by RFAM. Since we have a hierarchical clustering, different sets of clusters can be defined by cutting the tree at different thresholds ϑ, and we have to compare all these thresholds to find the set of clusters with the best agreement. The problem of comparing the partition defined by a given set of clusters (generated by cutting the tree at some specific level) with the partition defined by RFAM is now transformed into a classification problem as follows. We consider all possible pairs of sequences, and define the number of true positives (ss) as the number of sequence pairs from the same family that lie in the same cluster. Analogously, the number of false positives, false negatives, and true negatives are given by the number of pairs from different families but same cluster (ds), same family but different clusters (sd), and different families and different clusters (dd), respectively. Sensitivity and specificity are then defined as usual, namely *spec* = *dd*/(*dd* + *ds*) and *sens* = *ss*/(*ss* + *sd*). The receiver operating characteristic (ROC), obtained by plotting the sensitivity against the false positive rate (1-specificity) for different values of the cutoff ϑ, is shown in Figure 1.

A problem in the comparison with RFAM families is that different families exhibit very different diversity: some families consist only of closely related sequences while others accommodate significant variation in sequence and structure. Therefore, one should not expect that the RFAM family division can be modeled by using one fixed threshold ϑ for all families. We therefore consider a local, family-wise, criterion for the clustering quality. For a given RFAM family *R* and a cluster *C,* we define the recall *r*(*R*,*C*) as the fraction of members from *R* contained in *C*, i.e., *r*(*R*,*C*) = |*R*∩*C*|/|*R*|. For each family and a given minimum recall 0.5 < *r* ≤ 1, we can always determine the minimal threshold ϑ such that there is a unique cluster *C* with *r*(*R*,*C*) ≥ *r*. A measure of how well the clustering reconstructs the family *R* is then the associated precision *p*(*R*,*C*) = |*R*∩*C*|/|*C*|. An equal assessment of precision and recall is given with the F-measure:

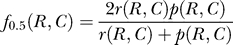



Averages are weighted by family size. Families that are only represented by one sequence do not contribute to the average as their precision is always 1.

Table 1 shows the average precision and F-measure weighted by family size for different minimum recall levels between 0.5 and 0.95. If we require that least 70% of a family (= minimal recall level) are grouped within the same cluster level, we get in fact on average a recall of 80%. In this case, we observe on average 32% false positive sequences within this cluster. Of course, we have much better values for some families such as 5S rRNA, where we have a precision of 100% at a recall level of 95%. The results also show that we are able to correctly cluster larger RNAs as well. For the members of the SSU_rRNA_5 family (accession code RF00177) included in our test set (recall that we have restricted the length to at most 400), 72.46% of them were clustered together in one single, pure cluster containing no other sequences. The sequences in this cluster have an average identity of 56.31%, and an average sequence length of 257. The complete RFAM tree constructed with our method is given in File Collection S1.

**Table 1:**
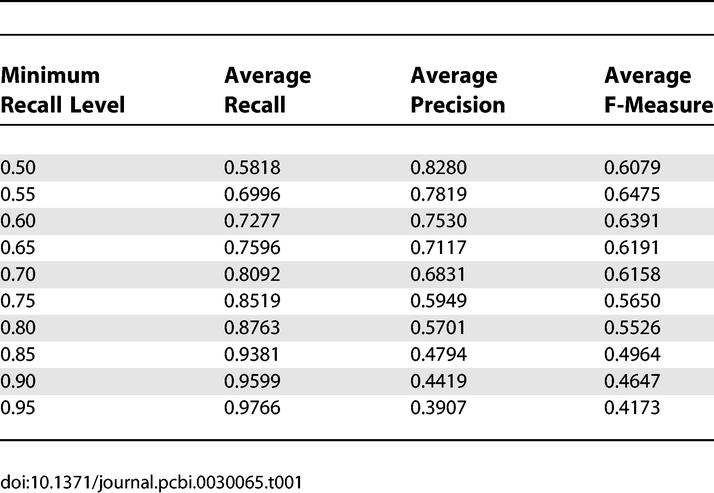
Average Precision and F-Measure for Different Minimum Recall Levels

Concerning the formation of classes comprising several families, this mainly makes sense for classes such as tRNAs and miRNAs which have a similar structure, but, for example, not for ribosomal RNAs where there are four structurally different families. The best classification is observed for the class of all tRNAs. They still have a precision of 96% at a recall level of 95%. Concerning the class of all miRNAs, they are (not surprisingly) grouped in several separate clusters. However, we have a large cluster comprising 85% of all 213 miRNAs and only 18% false positive sequences.

### Clustering of ncRNA Candidates in Gammaproteobacteria

An RNAz screen of six related gammaproteobacteria resulted in an ncRNA candidate set of 123 unique loci of the reference organism Escherichia coli. The screen follows the same pipeline as in [14,15] but includes a new approach to build multiple alignments. Only alignments with homolog sequences of at least three genomes, with maximal pairwise blast e-values of 1e-10 and a minimal length of 40 nt were retained for input to the RNAz pipeline.

**Figure 2:**
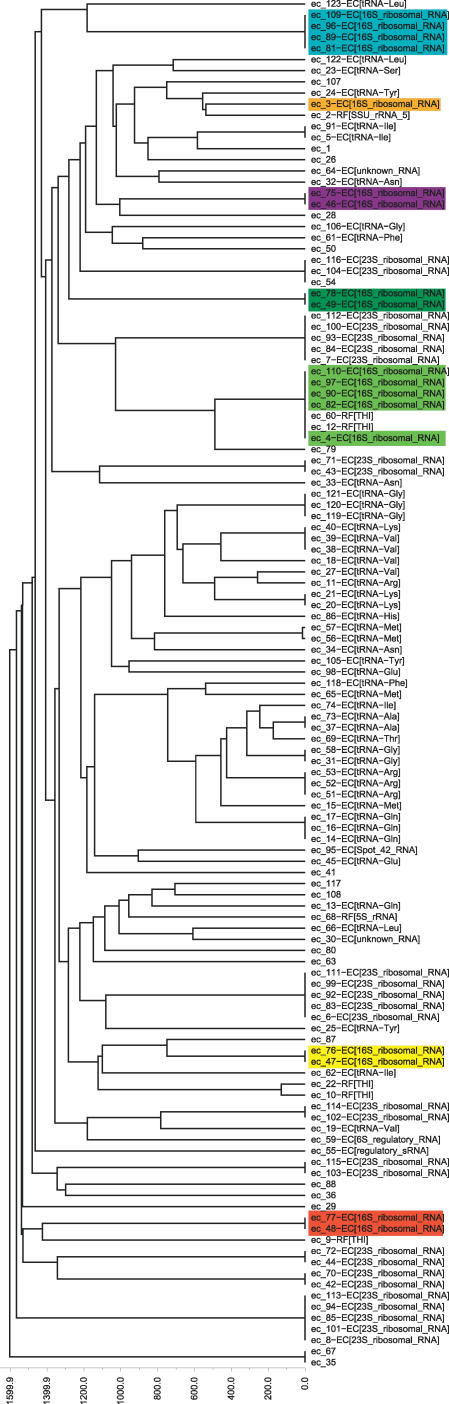
Complete WPGMA Clustering Tree for ncRNA Candidates in Gammaproteobacteria E. coli Candidates are annotated with known E. coli ncRNAs (*EC*[...]), or if such do not exist, then with ncRNAs from the RFAM database (*RF*[...]). The colored boxes correspond to different substructures of 16S as found by the RNAz screen. See Figure 3 for the location of these substructures. The situation is the same for 23S (see File Collection S1).

That the majority of ncRNA candidates could be annotated with known E. coli ncRNAs (labeled with *EC*[...] in Figure 2) is not surprising, as the screen was set up with a restrictive e-value for the initial blast search. Further, only candidates with homologs in at least three gammaproteobacteria genomes are reported. This provides us with a second ncRNA candidate set to validate the clustering approach, which in contrast to the RFAM seed sequences in the earlier section, Evaluation of the Clustering Procedure, was detected by RNAz. A candidate was annotated to be a known E. coli ncRNA if their genomic regions overlap to at least 70%. If such an annotation was not available, a blast search against the RFAM database (*E* < 1e-6) identified further homolog ncRNAs.

**Figure 3:**
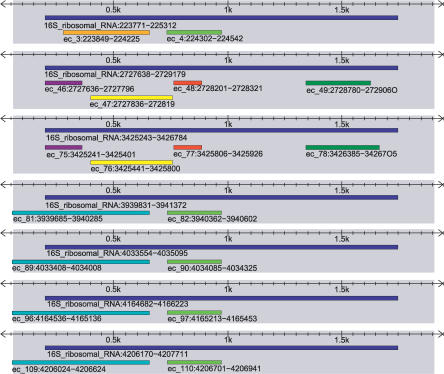
Locations of RNAz Predictions for 16S rRNA The dark blue box is the annotated 16S rRNA. The other boxes denote the RNAz predictions. Boxes with the same color are clustered together by our clustering procedure (see Figure 2). This shows that we correctly cluster the corresponding substructures.

In Figure 2, the complete WPGMA tree is depicted. It is nicely seen that again tRNAs get grouped in one separate cluster. Even tRNAs coding for the same amino acid are mostly found within the same subclusters.

At first glance, the distribution of rRNAs in Figure 2 is disappointing. Different families of rRNAs appear in several separate clusters; however, RNAz predictions for 16S and 23S do not fall into a single cluster. This distribution results from a shortcoming of the RNAz input screen rather than from a weakness of the clustering method. Since RNAz scores alignments in relatively short slices, large structured RNAs are in general not detected as a single contiguous locus. Rather, several substructures are recognized for both 16S and 23S RNAs, which to a certain extent depend on the exact location of sequence windows that are used for the RNAz scoring. As demonstrated in Figure 3 and in detail in File Collection S1, corresponding substructures (including features up to 800 nt in length) from the different rRNA loci in the E. coli genome are correctly clustered together.

### Clustering of ncRNA Candidates in C. intestinalis

**Figure 4:**
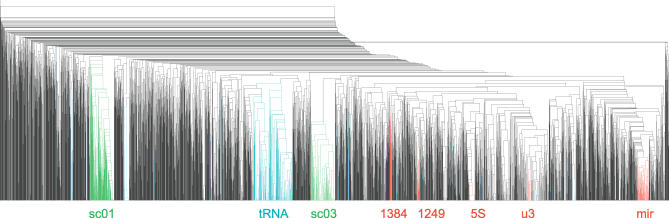
Summary of the Clustering Procedure The WPGMA tree contains 3,332 putative ncRNAs. A few large, prominent clusters are indicated. Among them are tRNAs and U3 snRNA, and an miRNA cluster, Figure 5, which contains the known miRNAs *mir-124-a/b* and *let-7* as well as candidates for *mir-126* and *mir-7*. Clusters 1384 in Figure 6 and 249 in Figure 7 are good candidates for novel ncRNA classes. sc01 and sc03 are both example clusters based on high sequence similarity.

The dataset resulting from the RNAz-based survey for conserved ncRNAs in the genomes of the ascidians C. intestinalis and C. savignyi [14] consists of 3,332 predicted structured RNAs, of which only about 500 could be annotated as members of well-known RNA families. The overwhelming majority of the known RNAs are the 301 tRNAs recognized by RNAz. Figure 4 summarizes the results of the clustering procedure.

**Figure 5:**
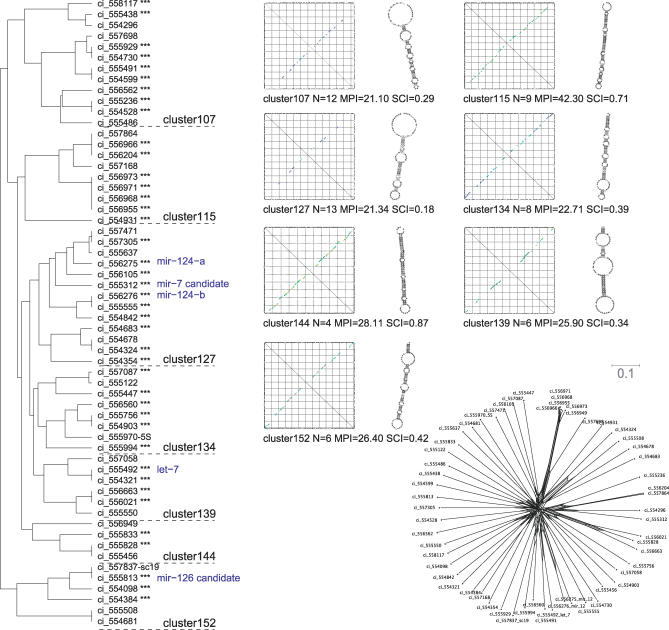
Cluster Containing Known and Predicted C. intestinalis microRNAs The two known *mir-124* paralogs are members of subcluster 127, whereas the known *let-7* is found in subcluster 139. Sequence ci_555813 in subcluster 152 contains a *mir-126* candidate (UCGUACCGUGAGUAAUAAAGC) and ci_555312 in subcluster 127 a *mir-7* candidate (UGGAAGACUAGUGAUUUUGUUGU). Forty of the 58 cluster members (marked with ***) are classified as putative microRNAs by RNAmicro [37]. The fourth known microRNA in urochordates, *mir-92,* does not fall into this structural cluster. Members of the cluster are not sequence related (NeighborNet in the bottom right corner). N, number of sequences in cluster.

**Figure 6:**
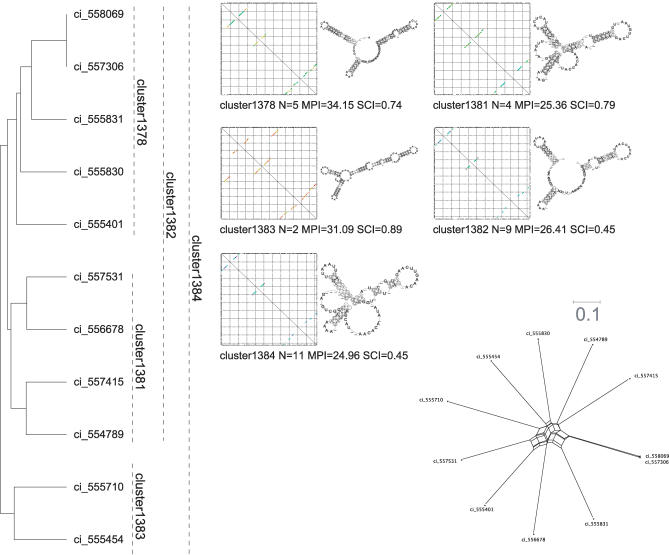
Cluster 1384 Groups Sequences with a Well-Conserved Secondary Structure Consisting of Three Stem Loops Whereas the sequence identity is low, we observe a high structural conservation. N, number of sequences in cluster.

**Figure 7:**
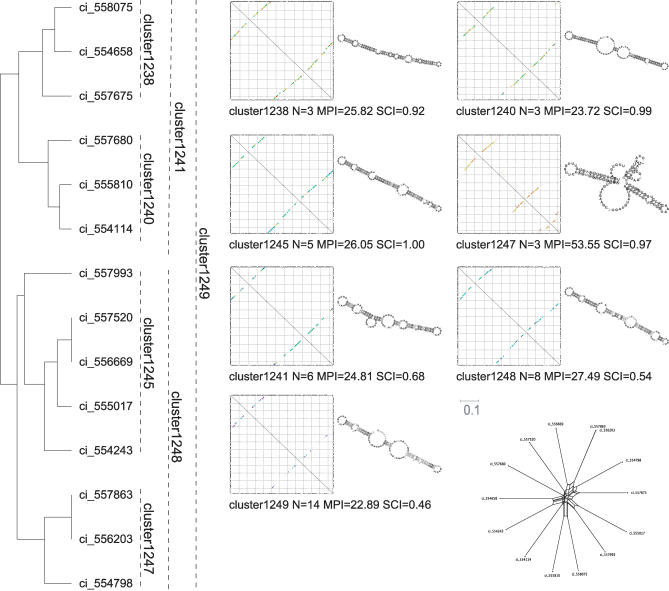
Example of Structure-Based Clustering of Very Diverse Sequences Which Might Form a Novel ncRNA Class The consensus structure models thus show a large number of compensatory mutations. N, number of sequences in cluster.

At first glance, the result might look disappointing as we find a large number of predictions that do not belong to any tight cluster. This is not surprising, however, given that we expect a very high noise level in this dataset. (1) The RNAz screen has an estimated false discovery rate of about 18%. (2) No attempts have been made to correct the fairly unreliable strand prediction of RNAz, which has an error rate up to 30% [32]. (3) We can expect that a significant fraction of structured elements have been predicted only partially. (4) Thermodynamic consensus structure predictions based on only pairwise alignments are far from perfect [19,33]. It is thus not surprising that only a fraction of the input data can be assigned to meaningful clusters.

As expected, the largest and most prominent cluster comprises tRNAs. As discussed in some detail in [34], this tRNA cluster is composed of subclusters corresponding to homologous tRNAs with common anticodons. Several other well-known multigene families are easily identifiable as structural clusters, including the U5 snRNAs, U3 snRNAs, and 5S rRNAs. Several families of multicopy genes with common secondary structure are present in the *Ciona* genomes [34]. Most of them are also readily identifiable in the structural cluster tree. Since these subclusters are already easily detectable on the sequence level, they are of little interest for the structured-based approach pursued here.

A more interesting example is a cluster, Figure 5, that contains two paralogs of *mir-124* and one copy of *let-7* microRNAs that were previously described in computational screens of C. intestinalis [35,36], as well as good candidates for *mir-126* and *mir-7*. The other members of the cluster have no sequence similarity with known microRNA families compiled in miRBase release 9.0 (blast *E* ≤ 0.001). Both *mir-124* candidates occur within introns of known mRNAs of C. intestinalis (JGI2.0), while *mir-126* and *mir-7* do not seem to be located in an intron. That a large cluster of known and putative miRNAs was detected demonstrates that annotation of ncRNA candidates is highly improved by structure-based clustering. The majority of cluster members could not be identified as miRNA candidates by sequence comparison alone [14]. Further, a comprehensive comparative screen for miRNAs across the metazoan species identified only a few homologs with high sequence similarity within the urochordates [36], raising the question if there may exist a group of yet unknown miRNA families within the urochordates.

Figures 6 and 7 highlight two novel clusters of structurally similar predictions for which no functional or class assignment is available. The neighbor-net graphs in the insets show the sequence distance within the example cluster. Since the sequence distance is on average larger than 0.5, this confirms that the clusters are defined essentially based on structural similarities. While our examples usually contain some subsets of related sequences, overall there is little or no detectable sequence conservation so that the clusters could not have been detected by sequence similarity alone. Since many ncRNAs, in particular snRNAs, tend to form multigene families (often evolving under some form of concerted evolution that keeps the family members nearly identical), a moderate copy number in the genome can be interpreted as supporting the hypothesis that the candidate is indeed a true ncRNA.

In cluster 1384, Figure 6, for example, sequences with a well-conserved secondary structure but low sequence similarity are grouped. Nine of 11 sequences of cluster 1384 could be exactly mapped to the new C. intestinalis assembly JGI2.0. The structural cluster contains three subclusters, 1378, 1381, and 1383, that have overall structural features in common. All subclusters have three stem loops originating from one single multiloop as consensus structure. But their length and number of internal loops differ. Their grouping into the superclusters 1382 and 1384 are justified by compensatory mutations. Two sequences of subcluster 1378 and one of subcluster 1381 appear within an intron of *Ciona*-mRNA AK113484. Whereas the two sequences of subcluster 1378 appear within the same copy of mRNA AK113484 on chr01p, the sequence in subcluster 1381 occurs in a copy on chr04q. Six different genomic copies of AK113484 exist in JGI2.0, but none of the intronic regions where the ncRNA candidates are found are associated with repeats. This allows the conclusion that those ncRNA candidates are indeed functional ncRNAs as their sequences are highly diverged, whereas they share common structural features and appear within the same *Ciona*-mRNA. One sequence of subcluster 1383 occurs in an exon of the known protein coding *Ciona*-mRNA AK114007. All other elements are intergenic or at least the corresponding mRNAs are not yet known.

Cluster 1249 is also composed of highly divergent sequences but similar secondary structures. Two sequences of subcluster 1247 appear within an intron of the *Ciona*-mRNA AK174830. Subclusters 1238 and 1245 contain one sequence occurring in an intron of *Ciona*-mRNA AK222260 and AK116291, respectively.

Clusters 1384 and 1249 are good candidates for novel classes of urochordate-specific ncRNAs, since none of the sequences has detectable ncRNA homologs in vertebrates.

## Discussion

Genome-wide studies, both experimental and computational, have uncovered tens of thousands of transcripts in higher Eukaryotes that have little or no protein-coding capacity. For a large subset of these, there is evidence for selection acting to preserve secondary-structure motifs. Many classes of functional RNAs, on the other hand, can be recognized based on structural similarities. It is thus natural to ask if the available data contain evidence for novel families and classes of structured RNAs, for which so far no representative has been well-characterized experimentally. To answer this question, it is necessary to cluster the candidate RNAs based on their structural features, a task that is computationally much harder than clustering based on sequence similarity.

We present here a new tool, LocARNA, which implements a novel, more efficient variant of the Sankoff algorithm. We have demonstrated that LocARNA is fast enough to make structure-based clustering of thousands of putative structured RNAs feasible. The main reason for its superior efficiency is due to the prefiltering of the base pairs by their probability, and an efficient computation scheme that is able to profit from the reduced number of base pairs considered. The method is also robust enough to find significant clusters in fairly noisy, realistic data that contain a substantial fraction of false positive predictions. We have successfully tested the tool on the sequences of the RFAM seed alignments.

The LocARNA implements a local sequence structure alignment method, which is required when applied to candidate ncRNA sequences where the exact region of interest is not exactly known (of course, the tool can also be applied to global alignment problems). Clearly, there is a length dependency in the scores, which has several sources, one being the calculation of pair probabilities. This influences both pairwise alignment and the clustering, which implies that the ncRNAs to be clustered should not diverge too much in length. This is the case in many applications like the clustering of predicted ncRNAs. A more precise treatment of the different kinds of dependencies (such as GC content) is planned for a future version.

Since perfect predictions and experimentally determined structures are not available, it is imperative to have a method that can identify clusters on *imperfect* structure prediction, although this also implies that we cannot hope for perfect pure clusters—some “contamination” and some sequences that fall elsewhere in the clustering are thus unavoidable. The ROC curve in Figure 1 shows that LocARNA indeed achieves this goal.

The application of the tool to a dataset of more than 3,000 predicted structured RNAs in urochordates showed that the clustering approach not only recovers known RNA families and classes such as tRNAs, but also predicts several candidates for novel ncRNA classes. In some cases we find that additional sequences are identified as structural relatives of known RNA families. In this way we have identified, for example, a *mir-126* and a *mir-7* homolog that were not detected in previous computational studies. More importantly, however, we also find structure-based clusters that are candidates for novel, presumably urochordate-specific, RNA classes. We find that these clusters often contain subclusters consisting of multicopy sequences. Comparing this with the characteristics of several well-studied ncRNA families, in particular tRNAs, the snRNAs associated with the major spliceosome, and SL RNAs, lends further credibility to the hypothesis that these sequences indeed form a bona fide RNA class.

## Supporting Information

File Collection S1 Supplemental Material, Zip File of html Directory http://www.bioinf.uni-freiburg.de/Supplements/locarna-06-12
(643 KB ZIP)

### Accession Numbers

Accession numbers starting with RF (e.g., RF00177) are taken from the RNA families database RFAM (http://www.sanger.ac.uk/Software/Rfam/) and denote different RNA families. Accession numbers starting with AK (e.g., AK113484) describe Ciona intestinalis mRNAs in the GenBank database (http://www.ncbi.nlm.nih.gov/entrez/query.fcgi?db=Nucleotide).

## Abbreviations

LocARNA;: local alignment of RNA
MFE: minimum free energy
MPI: mean pairwise sequence identity
ncRNA: noncoding RNA
ROC: receiver operating characteristic
SCI: structure conservation index
WPGMA: weighted pair group method algorithm
