# Supplementary material for: Inferring Noncoding RNA Families and Classes by Means of Genome-Scale Structure-Based Clustering
Source: PLoS Comput Biol. 2007 Apr 13;3(4):e65. doi: 10.1371/journal.pcbi.0030065 (PMC1851984; doi:10.1371/journal.pcbi.0030065)
Supplement: Supplementary file 1 [file pcbi.0030065.sd001.zip › locarna-06-12/index.html]

 <!--#echo var="globalTitleString" --> - Supplements


# Supplemental Material: Inferring Non-Coding RNA Families and Classes by Means of Structure-Based Clustering


 *Sebastian Will, Kristin Reiche, Ivo L. Hofacker,
Peter F. Stadler, Rolf Backofen* 

- Software LocARNA, release 0.99 [TarGz Package]
- Pipeline for extracting clusters [TarGz Package]
- Complete Tree of the 3332 Ciona intestinalis ncRNA candidate
  sequences in JGI 1.0 [PostScript][NEWICK Tree Format]\*
- Clustering of RFAM seed alignment sequences [PostScript][NEWICK Tree Format]\*
- Ciona intestinalis ncRNA candidates conserved in
  C. intestinalis and C. savignyi (JGI 1.0)   
   [Supplemental material of publication "Non-coding RNAs in Ciona intestinalis"]
- Ciona intestinalis ncRNA candidates mapped to assembly JGI 2.0
  (2544 of 3332 candidates exactly found in JGI 2.0)
   [Bed Format]
- Tree of known E.Coli ncRNAs [PostScript]
- mir-7 candidate ci\_555312
  - Trimmed alignment with known mir-7 miRNAs [PostScript]
  - Predicted secondary structure of ci\_555312 [PostScript]
- mir-126 candidate ci\_555813
  - Trimmed alignment with known mir-126 miRNAs [PostScript]
  - Predicted secondary structure of ci\_555813 [PostScript]
- Cluster 1384 (from Fig. 4)
  - Colored multiple alignment with consensus structure
    [PostScript]
  - RNAalifold drawing of consensus structure
    [PostScript]
  - Cluster 1249 (from Fig. 5)
    - Colored multiple alignment with consensus structure
      [PostScript]
    - RNAalifold drawing of consensus structure
      [PostScript]
    - Coverage of 16S and 23S rRNA in E.coli by the RNAz screen used for
      the tree of Fig. 6
        
      The figures show the ncRNA candidates of the screen that are annotated as 16S
      rRNA (23S rRNA, resp.) and how they are matched to the whole
      rRNA. NcRNA candidates in the same cluster are colored equally.
      - ncRNAs matching 16S rRNA [PostScript]
      - ncRNAs matching 23S rRNA [PostScript]

\* NEWICK tree file format can be viewed with njplot
